# Usefulness of 3D T1-SPACE in Combination With 3D-TOF MRA for Follow-Up Evaluation of Intracranial Aneurysms Treated With Pipeline Embolization Devices

**DOI:** 10.3389/fneur.2020.542493

**Published:** 2020-12-11

**Authors:** Qiuji Shao, Qiaowei Wu, Qiang Li, Tianxiao Li, Li Li, Kaitao Chang, Meiyun Wang

**Affiliations:** ^1^Department of Cerebrovascular Disease, Zhengzhou University People's Hospital, Henan Provincial People's Hospital and Henan University People's Hospital, Zhengzhou, China; ^2^Department of Radiology, Zhengzhou University People's Hospital, Henan Provincial People's Hospital and Henan University People's Hospital, Zhengzhou, China

**Keywords:** MRI, stent, follow-up, flow diverter, endovascular treatment, intracranial aneurysm

## Abstract

**Object:** Follow-up evaluation of intracranial aneurysms treated by flow-diverting stents using MRI is challenging due to the presence of imaging artifacts. This study evaluated 3D T1-SPACE in combination with 3D-TOF sequence for follow-up evaluation of intracranial aneurysms treated with Pipeline embolization devices.

**Methods:** Forty patients with 53 intracranial aneurysms who were treated with Pipeline Embolization Devices from October 2018 to July 2019 were enrolled in this study. All patients were evaluated for aneurysm occlusion and stent patency 4 to 7 months post-treatment using 3D T1-SPACE sequence, 3D-TOF MRA, and DSA examinations.

**Results:** With regards to aneurysm occlusion, the intermodality (DSA and 3D-TOF MRA) agreement was good (κ = 0.755). The specificity of 3D-TOF MRA was 94.4% (34/36), the sensitivity was 76.5% (13/17), the total coincidence rate was 88.7% (47/53). With regards to the patency of the stented arteries after PED treatment, 3D T1-SPACE sequence was more accurate compared to 3D-TOF MRA (Z = −6.283, *P* < 0.001), with a no-artifact rate of 95.7% (44/46).

**Conclusions:** 3D T1-SPACE sequence provides better image quality and higher accuracy for evaluating stented parent arteries compared to TOF-MRA. 3D-TOF MRA may be valuable in the evaluation of aneurysm occlusion. The combination of these two modalities may be used for long-term follow-up of intracranial aneurysms treated with Pipeline Embolization Devices.

## Introduction

Endovascular treatment for cerebral aneurysms has significantly changed from intrasaccular embolization to endovascular reconstruction and flow diversion (1). Pipeline Embolization Devices (PED) has gained widespread acceptance in treating intracranial aneurysms, especially large and giant internal carotid artery (ICA) aneurysms and has been demonstrated to be safe and effective in numerous clinical studies (2, 3). Some evidence-based studies have supported broadening the use of PED for the treatment of indications such as unruptured, small, and saccular aneurysms (<10 mm) (4, 5). Aneurysm occlusion after PED treatment is a progressive phenomenon within several months to years (6). Some patients may develop in-stent stenosis or artery occlusion. Hence, imaging follow-up evaluations after flow diversion (FD) treatment is essential.

DSA is the gold standard for follow-up evaluation of intracranial aneurysms. However, it is an invasive procedure with several risks, such as hematoma in the puncture site and thromboembolic complications. Furthermore, it is challenging for patients to undergo long-term repeated DSA examinations. Non-invasive follow-up evaluations using MR imaging is more commonly used for interventional therapy of intracranial aneurysms. These include 3D time of flight MRA (3D-TOF MRA) and contrast-enhanced MRA (CE-MRA). They have good sensitivity and accuracy for evaluating aneurysms treated using simple coil embolization (7). However, for patients treated with stent-assisted embolization, the MRA methods mentioned above inadequately display residual aneurysm and in-stent patency due to the presence of artifacts and radiofrequency shielding caused by the stents (8). In addition, it is very challenging for traditional MRA techniques to evaluate PED efficacy.

Three-dimensional T1-weighted sampling with application-optimized contrasts using different flip angle evolutions (3D T1-SPACE) provides three-dimensional, large-scale and high spatial resolution images of the intracranial vessel wall, and has demonstrated good results for intracranial vascular lesions (9). This study assessed the accuracy and effectiveness of the 3D T1-SPACE technique in combination with 3D-TOF MRA for follow-up evaluation of aneurysms treated using Pipeline-Flex Embolization Devices and compared the results to DSA.

## Materials and Methods

### Study Population

The study was approved by our local ethics board. From October 2018 to July 2019, a total of 40 patients with 53 aneurysms who were treated with PED were retrospectively enrolled in our study. The inclusion criteria were as follows: patients (aged 18–80 years) who had intracranial aneurysms treated with PED and obtained the two MR imaging techniques (3D-TOF MRA and 3D T1-SPACE) and DSA in the follow-up, both MRI sequences and DSA performed within 3 days apart. Informed consent was obtained from each patient prior to MRI examinations.

### Imaging Technique for MRI Scans

Both MRI sequences were performed on a 3.0 T Siemens Prisma MRI scanner (Siemens Medical, Germany) using a 64-channel head coil. The imaging parameters for 3D TOF-MRA images were as follows: TR, 21ms; TE, 3.45 ms; matrix, 320 × 232; field of view, 200 mm × 181 mm; section thickness, 0.60 mm; flip angle, 18°; total acquisition time, 3:36 min. Post-processing and analysis of images were performed using real-time 3D display technology on the Siemens Syngo workstations. Images were reconstructed using maximum intensity projection (MIP) and the aneurysm occlusion was observed using source images (SIs) by multi-planar reformat (MPR). For 3D T1-SPACE scanning parameters: TR, 900 ms; TE, 14 ms; matrix, 320 × 320; field of view, 170 mm × 170 mm; section thickness, 0.50 mm; number of sections, 224; scanning time, 8:29 min; The contrast agent was gadopentetate (Magen Weixian, Germany), and was intravenously injected at a dose of 0.2 mmol/kg. Three-dimensional thin-layer images were post-processed, and 3D T1-SPACE original images were imported into the Siemens workstations. Long and short axes of the vessels were processed based on the anatomical position of the two ends of PED. Presence of stenosis, occlusion or delayed thrombosis in the stent of the parent artery was then evaluated.

### Imaging Technique for DSA

Catheterization was performed using a modified Seldinger technique through the femoral artery under local anesthesia and 3D rotational angiography of the target vessels (FD20, Philips, Netherlands) was performed. After selecting the optimal projection angle of the aneurysm based on the 3D post-processing system of the DSA device, the target lesions were enlarged for 2D angiography to determine whether the aneurysm sac or neck was residual, as well as whether there was patency in the parent vessel.

### Image Analysis

According to the O'kelly-Marotta (OKM) classification (10), the radiological results of the aneurysm residual is graded as: A—complete (>95%); B—incomplete (5–95%); C—neck remnant (<5%); or D—no filling (0%). In-stent lumen of the parent artery was evaluated using a 4-point scale as follows: (1) not visible (almost no signal in the stent); (2) poor (structures are slightly visible but with significant blurring or artifacts, not diagnostic); (3) good (good quality diagnostic information with minimal blurring or artifacts); (4) excellent (excellent-quality diagnostic information, the shape of depiction is nearly equal to that of DSA) (11). Magnetic resonance and DSA images were independently reviewed by two interventional neuroradiologists (both with >10 years' experience). When a different reading was proposed, consensus was found after an intensive discussion between the two radiologists. 3D-TOF MRA, 3D T1-SPACE, and DSA were evaluated separately without knowledge of the MRA or DSA examination results. The location of the aneurysms to be evaluated was provided to the readers.

### Statistical Analysis

All statistical analyses were performed using SPSS 22.0 software (IBM SPSS Inc., Chicago, IL, USA). Quantitative variables were described as mean ± SD or median (interquartile range) and qualitative variables were described as numbers and percentages. The κ statistic was used to analyze the aneurysm occlusion between DSA and 3D TOF-MRA, and Wilcoxon singed rank test was used to analyze the subjective scores for the quality of parent artery images. *P* < 0.05 were considered statistically significant. DSA was used as the reference standard to calculate the specificity and total coincidence rate of the aneurysm occlusion degree evaluated by 3D TOF-MRA.

## Results

### Patient and Aneurysm Characteristics

A total of 40 patients with 53 aneurysms underwent imaging follow-up evaluations within a mean period of 6 ± 0.8 months. Of the 40 patients, 30 (75%) had one aneurysm, 7 (17.5%) had two aneurysms, and 3 (7.5%) had three aneurysms. A total of 46 PEDs were used. Thirty-three patients (82.5%) with 37 aneurysms were treated using a single PED for one aneurysm, two patients (5%) with large aneurysms were treated with double overlapping PEDs for one aneurysm and 5 patients (12.5%) with tandem aneurysms were treated with a single PED. All aneurysms were unruptured. Forty-two aneurysms were treated exclusively with PED and 11 with coils and PEDs (Table 1). None of the patients had perioperative complications.

**Table 1:** Patient and aneurysm characteristics.

**Characteristics**	
Mean age (range) (yr)	53.8 ± 10.4 (31–73)^a^
Female	31 (77.5%)
**Location**	
Internal carotid artery	46 (86.8%)
Posterior communicating artery	2 (3.8%)
Middle cerebral artery	3 (5.6%)
Vertebral artery	2 (3.8%)
**Aneurysm size**	
<5 mm	27 (51.0%)
5–10 mm	16 (30.2%)
11–25 mm	7 (13.2%)
>25 mm	3 (5.6%)
Follow-up (months)	6 (6, 6.75)^b^
Interval between DSA and MRA (days)	2 (1, 2)^b^

a *Mean ± standard deviation*.

b *Median with interquartile range*.

### Aneurysm Occlusion

The results for aneurysm occlusion using DSA and 3D TOF-MRA are shown in Table 2. Inconsistent results were observed for six aneurysms between these two methods. Two micro-small aneurysms were assessed as grade B by DSA, while 3D TOF-MRA showed complete occlusion. Two aneurysms were evaluated as grade C by DSA while no residual aneurysms were observed by 3D-TOF MRA. All four cases mentioned above had obvious slow flow determined by DSA. Complete occlusion was observed for two aneurysms by DSA, however 3D-TOF MRA showed high signals in the aneurysm sac (may have been due to metal artifacts caused by the coils), and hence was mistakenly classified as grade B.

**Table 2:** Evaluation of aneurysm occlusion using DSA and 3D-TOF MRA.

	**DSA**			
	**Grade A**	**Grade B**	**Grade C**	**Grade D**
**3D-TOF MRA**				
Grade A	0	0	0	0
Grade B	0	9	0	2
Grade C	0	0	4	0
Grade D	0	2	2	34
κ	κ = 0.755			

With regards to aneurysm occlusion, the intermodality (DSA and 3D-TOF MRA) agreement was good (κ = 0.755). Compared to DSA, the specificity of 3D-TOF MRA for evaluating aneurysm occlusion was 94.4% (34/36) the sensitivity was 76.5% (13/17), and the total coincidence rate was 88.7% (47/53).

### Stent Patency

**Figure 1:**
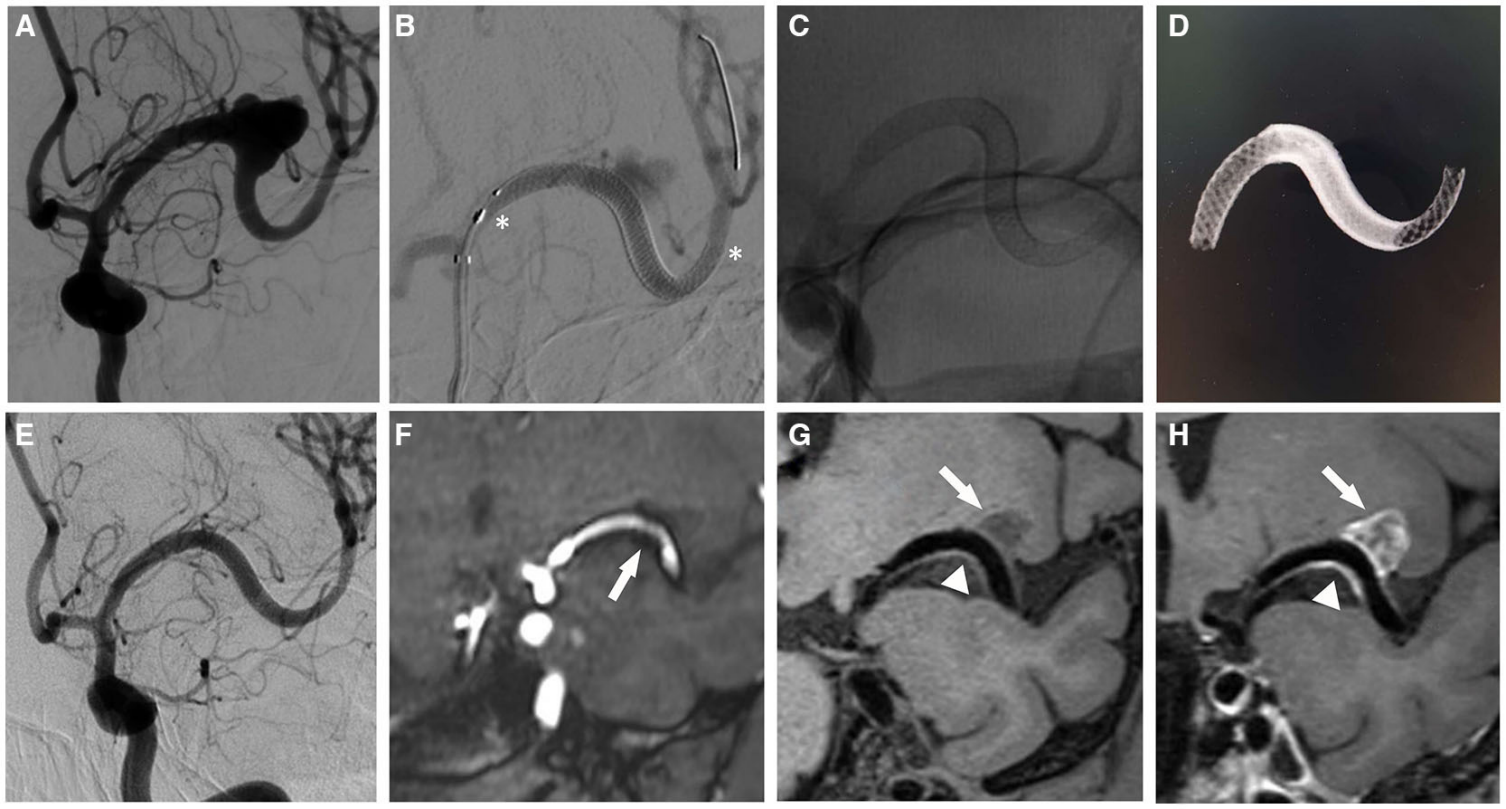
A 44 year-old woman with a middle cerebral artery aneurysm. **(A)** DSA shows the large irregular wide-necked aneurysm of the left middle cerebral artery M1 segment. **(B)** Cerebral angiography demonstrates the dual overlapping Pipeline Flex embolization devices (asterisk). **(C,D)** no subtraction angiography **(C)** and reconstruction of Vaso CT **(D)** performed immediately after the PED treatment shows the morphology and position of the stents. **(E)** the aneurysm completely occluded at 6 month follow-up. **(F)** the 3D-TOF MRA sequence also reveals a total occlusion of the aneurysm, however there was artifactual luminal narrowing of the stented parent artery (arrow). **(G)** the 3D T1-SPACE sequence demonstrates that excellent-quality diagnostic information of in-stent lumen without metal artifacts (arrowhead). **(H)** Enhanced scanning shows mixed signal intensity in the aneurysm sac (arrow), which is considered to the thrombus composition.

**Figure 2:**
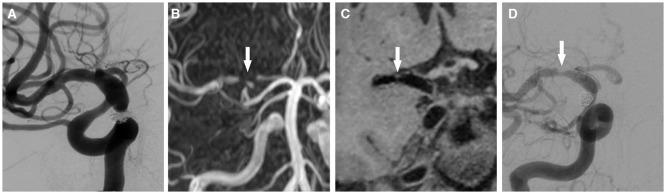
A 46 year-old man with a right posterior communicating artery aneurysm. **(A)** PED and coils was performed for it. **(B)** after 6 months, 3D-TOF MRA shows signal loss of the right proximal middle cerebral artery (false occlusion, arrow) and signal intensity of distal vessels. **(C)** the 3D T1-SPACE sequence demonstrates the moderate stenosis of the right origin segment of middle cerebral artery (arrow). **(D)** this result is consistent with DSA in evaluating of stent patency (arrow).

With regards to the patency of the stented arteries after PED treatment, 3 D T1-SPACE sequence was more accurate compared to 3D-TOF MRA (Z = −6.283, *P* < 0.001), with a no-artifact rate of 95.7% (44/46) (Figure 1, Table 3). Using the 3D T1-SPACE technique, all cases were evaluable and in-stent stenosis was observed in 5/46 stents (10.9%). This was consistent with the DSA results (Figure 2). Two of the five patients with parent vessel stenosis had a post-operative stenosis rate of 80% and 50%. The other 3 cases had preoperative parent artery stenosis, which improved after the procedure. None of these patients had neurological symptoms.

**Table 3:** Stented parent arteries evaluation using 3D T1-SPACE and 3D-TOF MRA.

**Methods**	***n***	**Unevaluable**		**Evaluable**	
		**Grade 1**	**Grade 2**	**Grade 3**	**Grade 4**
3DT1-SPACE	46	0	0	2	44
3D-TOF	46	1	7	38	0

*SPACE, sampling perfection with application-optimized contrasts by using different flip angle evolutions; TOF, time-of-flight*.

## Discussion

PED can promote thrombosis of the aneurysm sac and endothelialization of the parent artery. Cerebral aneurysms are cured anatomically and physiologically. Based on the embolization mechanism of FD, complete occlusion of intracranial aneurysms after PED treatment is time-dependent. In-stent stenosis could occur in certain patients during mid to long term follow-up after FD implantation. This is especially true for patients with poor vascular conditions, poor compliance to antiplatelet therapy, and poor management of the risk factors for atherosclerosis. John et al. (12) defined in-stent stenosis as intimal hyperplasia beyond the limits (>25%) of the metallic mesh and this study showed that PED was associated with a 9.8% rate of in-stent stenosis observed at a median follow-up period of 6 months. Hence, regular imaging follow-up evaluations after PED treatment is necessary to monitor aneurysm occlusion and stent patency.

With regards to the follow-up evaluation of aneurysm occlusion after PED implantation, the total coincidence rate of 3D-TOF MRA was 88.7% (47/53) compared with DSA. Thrombus formed by the coils after embolization had distinctly lower signals in 3D-TOF MRA, and was clearly distinguishable from the high signals of blood flow in the parent artery or the residual aneurysm sac. Hence, this imaging technique has a merit to detect residual flow or aneurysm recurrence. However, on 3D-TOF MRA, the metal artifacts of the flow-diverting stent and its magnetic shielding effect caused a false observation of stenosis or occlusion (Table 3). Conversely, in our preliminary study, the coils and thrombus in the aneurysm sac were observed as a low-medium mixed signal intensity by 3D T1-SPACE sequence. It contained various factors such as thrombus organization, fibrosis, and coil-related low signal intensities. However, blood flow also showed low signal intensity on this sequence. Thus, 3D T1-SPACE was unavailable to evaluate residual aneurysm lumen. By analyzing the SIs of the parent artery and the axial reconstructed images, the in-stent lumen was clearly visible in black blood 3D T1-SPACE sequence, with a no-artifact rate of 95.7% (44/46). This study demonstrated that 3D T1-SPACE had significant advantages for evaluating the patency of stented arteries after FD therapy. Hence, we demonstrated the utility of 3D-TOF MRA for follow-up evaluation of aneurysm occlusion and 3D T1-SPACE sequence for the parent artery after PED treatment.

At present, 3D-TOF MRA is the most frequently used non-invasive follow-up method for evaluating interventional embolization of intracranial aneurysms (13). For postoperative follow-up of patients who underwent simple coil for the treatment of intracranial aneurysm, 3.0T 3D-TOF MRA has high accuracy and specificity for detecting aneurysm remnants. However, for patients who underwent stent-assisted embolization, the magnetic and radiofrequency shielding causes a signal loss in the stent implantation area and manifests as a false in-stent stenosis or interruption (14). Takayama et al. (15) found that 3.0T 3D-TOF MRA was difficult for evaluating parent artery patency due to the influence of the stent on the magnetic field. Similarly, Thamburaj et al. (16) demonstrated that the bright blood 3D-TOF sequence had a false positive rate of 55–60% for evaluating parent vessels.

CE-MRA is a commonly used contrast-enhanced MRI angiography technique that provides a robust blood flow signal using gadolinium contrast agents. It can identify slow residual flow of recurrent aneurysms. This imaging technique improves the detection sensitivity of the residual sac and reduces the metal artifacts associated with the stent to a certain extent (17). However, a meta-analysis study performed by van Amerongen et al. (18) did not demonstrate better results compared to 3D-TOF MRA. Marciano et al. (8) demonstrated that the accuracy of 3.0T TOF-MRA and CE-MRA was not high for evaluating remnant aneurysms and parent artery patency in patients with stent-assisted coil embolization. In addition, Irie et al. (11) using silent MRA to assess patients with aneurysms after stent-assisted embolization showed that it was superior to 3D-TOF MRA for determining in-stent blood flow signals. However, a certain degree of false positives was observed when evaluating stent patency.

PED provides flow diversion using an attenuated braided bimetallic design of 75% cobalt-chromium and 25% platinum tungsten and has a 30 to 35% metal surface area. It could induce magnetic susceptibility artifacts, which consequently reduces the accuracy of traditional MRAs to evaluate the parent artery (19). Studies on non-invasive imaging follow-up techniques after PED treatment are limited (20–22). Patzig et al. (21) demonstrated that CE-MRA was invaluable when it was used to evaluate residual aneurysm after PED treatment. However, it has limitations when used to assess the in-stent lumen. Attali et al. (20) showed that CE-MRA was superior to 3D-TOF MRA in evaluating aneurysm occlusion after PED therapy, but had poor results when used to evaluate stent patency. Similarly, Tsay et al. (22) demonstrated that CE-MRA could lead to false-negative results when used for long-term imaging follow-up evaluations. CE-MRA in combination with other multi-imaging modalities is essential. Imaging follow-ups using traditional MRA techniques to evaluate intracranial aneurysms treated with PED is challenging. Reducing the metal artifacts of the stents, as well as improving image quality and accuracy of MR angiographs to evaluate such patients was the focus of the current study.

High-resolution magnetic resonance imaging has been increasingly used for the diagnosis and treatment of cerebrovascular diseases, i.e., for investigating the characteristics of intracranial vascular plaques and predicting the bleeding risk of unruptured aneurysms (23, 24). Park et al. (25) demonstrated a significant consistency between 3D HR-MRI and DSA for evaluating the nature and degree of intracranial arterial stenosis. Texakalidis et al. (26) showed that enhancement of the aneurysmal wall was associated with aneurysm rupture. This may be an effective non-invasive imaging method for assessing the risk of aneurysm rupture. Black blood MR imaging can clearly display the vascular wall as low signals by reducing the blood flow effect (27). Compared to HR-MRI technology that had limited scanning ranges and long image acquisition times, 3D T1-SPACE uses isotropic volume scanning for 3D high-resolution reconstruction at any level. It has the advantages of wide coverage and short scanning times and is capable of panoramic imaging of the intracranial artery wall. Hence, this study used 3D T1-SPACE sequence to evaluate the parent vessel after PED treatment.

This study has several limitations. Firstly, the patient cohort size was small, our results should be validated using larger multicenter patient cohorts with longer follow-up periods. Second, in most cases, follow-up MR imaging and DSA examinations were not performed on the same day. Hence, these two different modalities may not reflect the exact conditions in terms of the state of aneurysms and stented parent arteries. Third, as determined from this study, we observed that the evaluation of 3D-TOF MRA for partial slow blood flow of intracranial aneurysm after PED treatment needs to be further improved. Different vascular imaging modalities can be directly contrasted to evaluate the aneurysm after FD therapy in future studies. Fourth, due to motion artifacts, there were 2 cases that had poor image quality (using 3D T1-SPACE technique) for evaluating stented parent arteries. Hence, the image acquisition time of the black blood sequence should be reduced.

## Conclusion

According to the results of our study, 3D T1-SPACE sequence provides better image quality and higher accuracy for evaluating stented parent arteries compared to TOF-MRA. 3D-TOF MRA may be valuable in the evaluation of aneurysm occlusion. The combination of these two modalities may be used as a routine non-invasive follow-up evaluation method after PED treatment.

## Data Availability Statement

All data generated or analyzed during this study are included in this article, and unpublished data are available upon reasonable request from the corresponding author.

## Ethics Statement

The studies involving human participants were reviewed and approved by the Ethics Committee of Henan provincial people's hospital. The patients provided their written informed consent to participate in the study. Written informed consent was obtained from the individual(s) for the publication of any potentially identifiable images or data included in this article.

## Author Contributions

QS and QW contributed to study conception and design and drafted the manuscript. QS, QW, TL, LL, and QL contributed to data acquisition, data interpretation, and analysis. QS, QW, TL, and MW critically revised the paper and approved the final version of the manuscript. QL, KC, and LL contributed significant intellectual content. All authors made a significant contribution to the study and manuscript preparation and read and approved the final manuscript.

## Conflict of Interest

The authors declare that the research was conducted in the absence of any commercial or financial relationships that could be construed as a potential conflict of interest.

## Abbreviations

3D T1-SPACE: three-dimensional T1-weighted sampling with application-optimized contrasts using different flip angle evolutions
3D-TOF-MRA: three-dimensional time-of-flight magnetic resonance angiography
CE-MRA: contrast-enhanced MRA
PED: Pipeline Embolization Devices
FD: flow diversion
SIs: source images.
